# Draft Genome Sequence of Campylobacter jejuni ST-508 Strain Shizu21005, Isolated from an Asymptomatic Food Handler in Japan, 2021

**DOI:** 10.1128/mra.00316-22

**Published:** 2022-05-12

**Authors:** Aya Ogawa, Hiromi Nagaoka, Hiroshi Asakura

**Affiliations:** a Department of Microbiology, Shizuoka Institute of Environment and Hygiene, Shizuoka, Japan; b Division of Biomedical Food Research, National Institutes of Health Sciences, Kanagawa, Japan; Indiana University, Bloomington

## Abstract

Here, we report a draft genome sequence of Campylobacter jejuni strain Shizu21005, isolated from a food handler with no symptoms in Japan on March 2021. Its genome size was 1,656,785 bp, with 2 rRNAs, 35 tRNAs, and a coverage of 330×.

## ANNOUNCEMENT

Campylobacter jejuni and Campylobacter coli are two of the leading causes of foodborne gastroenteritis and are recognized to be transmitted mainly from poultry meat worldwide (1, 2). In Japan, a total of 154 foodborne campylobacteriosis cases has been reported, which represented 20.2% of the overall annual foodborne cases (764 cases) in 2021 (3). Similar to western countries, a recent epidemiological study has mounted evidence that poultry meats are one of the main sources for human campylobacteriosis in Japan (4). In March 2021, we surveyed the prevalence of C. jejuni and/or C. coli in the feces of food handlers working at a restaurant. Finally, C. jejuni was isolated from one asymptomatic food handler who engaged in cooking chicken meat products (designated strain Shizu21005). To obtain the isolate, one loop of the fecal sample from the food handler was incubated in 10 mL of Preston broth (Kanto Chemical, Tokyo, Japan) at 42°C for 24 h under microaerophilic conditions using the Anaeropack system (Mitsubishi Gas Chemicals, Tokyo, Japan). A total of 50 μL of the enriched culture was then spread onto modified charcoal-cefoperazone-deoxycholate agar (mCCDA) (Kanto Chemical) and incubated microaerobically at 42°C for 48 h as mentioned above. Five suspected colonies were subjected to the confirmation test according to ISO 10272-1:2017 (5) and finally identified to be C. jejuni. As the human sample was used for public health surveillance by the local government and detailed human ethics were not included here, we did not need to obtain independent permissions/approvals from our research organizations under our regulation. Although this pathogen was not recovered from the cooked products, we then attempted to collect its draft genome sequence data to characterize the obtained C. jejuni isolate. The C. jejuni strain Shizu21005 was grown on Mueller-Hinton agar (Becton Dickinson, Franklin Lakes, NJ) under microaerophilic conditions (85% N_2_, 5% O_2_, and 10% CO_2_) using a humidified multigas incubator (PHCbi, Tokyo, Japan) for 24 h. Genomic DNA was then extracted from C. jejuni Shizu21005 by using a Maxwell Rapid Sample Concentrator (RSC) blood DNA kit (Promega, Fitchburg, WI) according to the manufacturer’s instructions. The genomic DNA was used to construct a library using the Ion Xpress plus fragment library kit (Thermo Fischer Scientific, Waltham, MA) accordingly. After purification with E-gel (Thermo Fisher Scientific) and AMPureXP (Beckman Coulter, Brea, CA, USA) and quantification with the ion library Taqman quantitation kit (Thermo Fisher Scientific), the library (50 pM) was sequenced using an Ion 530 chip and Ion 510 & Ion 520 & Ion 530 kit Chef (Thermo Fisher Scientific) in single-end sequencing with an Ion Torrent GeneStudio S5 sequencer with Ion Chef (Thermo Fisher Scientific), resulting in a coverage of 330× with an average read length of 239 bases. The sequence reads consisted of 545.9 million bases that were trimmed and *de novo* assembled using the CLC Genomics Workbench v. 21 (Qiagen, Hilden, Germany). The parameters for trimming were as follows: ambiguous limit, 2; quality limit, 0.05; number of 5′-terminal nucleotides, 20; and number of 3′-terminal nucleotides, 5. The parameters for the *de novo* assembly were as follows: mapping mode, create simple contig sequences (fast); bubble size, 50; word size, 21; minimum contig length, 1,000 bp; perform scaffolding, no; and autodetect paired distances, yes. The draft genome sequence of the C. jejuni Shizu21005 was assembled into 80 contigs with an accumulated length of 1,656,785 bp (*N*_50_, 29,569 bp) and an average G+C content of 30.4%. The genome was annotated by the DFAST program (https://dfast.ddbj.nig.ac.jp/) (6) under default parameters (accessed on 12 March 2022). Annotation of the assembly identified 1,834 coding sequences (CDSs), 2 rRNAs, and 35 tRNAs.

The *in silico* multilocus sequence type (MLST) approach using the MLST 2.0 software (https://cge.cbs.dtu.dk/services/MLST/) (7) under default parameters (accessed on 12 March 2022) assigned the Shizu21005 strain to be sequence type 508 (ST-508), which was isolated previously from humans (8, 9) and animals, such as cattle and poultry (10, 11), in the United Kingdom and the United States. Meanwhile, its prevalence rates seemed to be rare regardless of the isolation source, and no reports existed for the isolation of ST-508 in Asian countries, including Japan, based on the Campylobacter MLST database (https://pubmlst.org/campylobacter, accessed on 14 March 2021).

A recent study showed no apparent evidence for host specialization of the ST-508 clonal complex (12), and thus, the virulence of ST-508 remains unclear. The whole-genome sequencing data shown here would be contributable to accumulate the bacterial genomic data in combination with their epidemiological background information, leading to an evaluation of the bacterial host specialization and virulence property in future work.

### Data availability.

The whole-genome project was deposited in GenBank with Sequence Read Archive (SRA) accession number DRR352099, BioProject accession number PRJDB13151, BioSample accession number SAMD00445926, and GenBank accession number CP087980. The version described in this paper is the first version, DRR352099.1.
